# Photosynthetic traits of *Phragmites australis* along an ecological gradient and developmental stages

**DOI:** 10.3389/fpls.2024.1476142

**Published:** 2025-01-08

**Authors:** Viktor R. Tóth

**Affiliations:** ^1^ Aquatic Botany and Microbial Ecology Research Group, Hungarian Research Network (HUN-REN) Balaton Limnological Research Institute, Tihany, Hungary; ^2^ National Laboratory for Water Science and Water Security, Hungarian Research Network (HUN-REN) Balaton Limnological Research Institute, Tihany, Hungary

**Keywords:** phenotypic plasticity, *Phragmites* degradation, reed photophysiology, trait variability, wetlands

## Abstract

Common reed (*Phragmites australis*) is a cosmopolitan species, though its dieback is a worldwide phenomenon. In order to assess the evolutionary role of phenotypic plasticity in a successful plant, the values and plasticity of photophysiological traits of *Phragmites australis* were investigated in the Lake Fertő wetlands at 5 sites with different degrees of reed degradation and along a seasonal sequence. On the one hand, along the established ecological degradation gradient, photophysiological traits of *Phragmites* changed significantly, affecting plant productivity, although no consistent gradient-type trends were observed. Gradual changes within a season in the values of photosynthetic traits were observed that were recorded in both degraded and stable stands, suggesting a universal response to seasonally changing environmental conditions that could not be overridden by the ecological gradient. On the other hand, reed plants exposed to different levels of degradation showed comparable physiological plasticity; there was no difference in trait variability between stable and degraded stands. This relatively uniform plasticity is likely to contribute to the resilience of reed plants by providing a wider range of adaptive traits under different conditions. In contrast, the 150-200% gradual change in photophysiological trait plasticity with senescence in *Phragmites* was also demonstrated, reflecting a more dynamic response of the photosynthetic apparatus to seasonal changes. Senescence affected the plasticity of plant traits independently of their degradation status, suggesting a more universal nature of seasonal changes. This research shows that under conditions of conservative resource use determined by stressful habitats, trait values respond to conditions, while trait plasticity shows minimal changes. Furthermore, phenological sequence significantly influenced both the values and the plasticity of the photosynthetic traits studied. Our results underline the impact of ecological degradation on reed physiology and highlight the importance of understanding both trait values and plasticity in plant responses to environmental and seasonal change.

## 1 Introduction

The natural world exhibits considerable temporal and spatial variability, and in order to be successful organisms must evolve the ability to adapt to these changing conditions. Common reed (*Phragmites australis* (Cav.) Trin ex Steud – referred to in this text as *Phragmites* and reed), is a widespread perennial emergent aquatic plant that can dominate vast areas by forming extensive monocultures in its habitats and can be found in wetland and aquatic habitats around the world. Its cosmopolitan distribution highlights its remarkable ability to acclimate and adapt to a wide range of environmental conditions, from pristine wetlands to anthropogenically altered ecosystems. *Phragmites* not only possesses a wide range of competitive traits (Kettenring et al., 2012; Eller et al., 2014), but also a significant plasticity of these traits that determines the success of the species under very different environmental conditions (Vretare et al., 2001; Mozdzer and Zieman, 2010; Eller et al., 2017). This success of the common reed in different habitats underlines the importance of understanding the trait variability of successful species in relation to environmental gradients and developmental stages (Clevering and Lissner, 1999; Meyerson et al., 2016; Eller et al., 2017), thus making it an interesting subject to study the adaptability of plants as well as facilitating its effective conservation and management.

While genetic diversity remains the key component of biodiversity, structuring, functioning and stabilising ecosystems (Salo and Gustafsson, 2016; Carvalho et al., 2019), knowledge of the effects of phenotypic plasticity (trait variability) on growth and functioning of wetland plants remains limited (Chambers et al., 2008). The plastic response of *Phragmites* to various extreme conditions serves to increase the success of the species (Clevering et al., 2001; Vretare et al., 2001; Engloner, 2009), although the functional value of this plasticity, its evolutionary role, has not been fully explored and thus understood. The importance and role of phenotypic plasticity in organismal adaptation to environmental change is probably related to its dynamic nature. Researchers have investigated various mechanisms underlying phenotypic plasticity, and experimental evidence has shown that phenotypic plasticity can enable organisms to adjust their morphology and physiology in response to environmental cues, thereby enhancing their fitness and survival (Ghalambor et al., 2007; Valladares et al., 2007; Nicotra et al., 2010).

Although phenotypic plasticity is intrinsic to organisms, it is not static and can be modulated by various factors. Research has shown that phenotypic plasticity can change over time, even within weeks, with factors such as senescence or seasonal variation influencing its expression (Nicotra et al., 2010; Stotz et al., 2021). Studies have shown that as organisms age, their capacity for phenotypic plasticity may decrease due to physiological changes or reduced environmental sensitivity. In addition, phenotypic plasticity can vary within a year in response to seasonal cues such as changes in temperature, photoperiod or resource availability (Nicotra et al., 2010; Stotz et al., 2021). These environmental fluctuations can trigger phenotypic adaptations, resulting in temporal shifts in the expression of plastic traits. Thus, phenotypic plasticity exhibits a dynamism that is shaped by both intrinsic and extrinsic factors, highlighting its adaptability in facilitating organismal responses to changing environmental conditions.

Studying functional traits and their plasticity in an otherwise successful plant could be useful in unravelling the mechanisms behind its adaptability and ecological success (Ackerly et al., 2000; Reich et al., 2003). Photosynthesis is a fundamental physiological process that directly influences plant growth and productivity, and patterns of intraspecific trait variation could provide valuable insights into the role of photosynthetic traits in evolutionary adaptation (Arntz and Delph, 2001; Maire et al., 2015). By studying how photosynthetic traits vary along ecological gradients and across phenological sequences, we can gain insights into the adaptive strategies used by populations to cope with different environmental challenges (McKown et al., 2013; Fajardo and Siefert, 2016).

Understanding the dynamics of photosynthetic traits in *Phragmites australis* populations with different ecological and phenological backgrounds contributes to the understanding of how divergence in photosynthetic traits could lead to evolutionary advantages for species and, consequently, have broader ecological and conservation goals (Lessmann et al., 2001; Mészáros et al., 2003; Tóth, 2016). Common reed has been shown to be able to adapt to very different environments by maintaining a higher photosynthetic capacity in different habitats compared to other species (Lessmann et al., 2001; Engloner, 2009; Eller et al., 2017). This higher rate allows reeds to fix more carbon, giving them a significant advantage in terms of biomass production and species expansion. Chlorophyll fluorescence techniques are convenient, fast and important tools in plant physiology studies, as they provide a non-invasive way to monitor the photosynthetic performance of plants (Roháček et al., 2008; Kalaji et al., 2016; Tóth, 2018; Tóth et al., 2019). As an early indicator of stress manifestation in plants, it can be used to determine and understand heterogeneity in leaf photochemical efficiency (Li et al., 2004; Stratoulias et al., 2015; Tóth, 2016, 2018) and can provide useful information on leaf photosynthetic performance.

The phenomenon of reed die-back has been observed across numerous lakes throughout Europe, with over 35 cases documented (Ostendorp, 1989; van der Putten, 1997), though some areas of North America experiencing similar phenomena (Reed and Cahoon, 1992; Visser et al., 1999). The die-back was primarily attributed to human interventions that altered the natural environments of these aquatic ecosystems. The common reed has experienced large-scale declines, especially in areas where hydrological regimes, water quality, or land use have been modified by anthropogenic activities. The most pronounced impacts have been observed in lakes that have undergone water-level regulation, eutrophication, and habitat fragmentation, with reed beds breaking up, reduced vitality, and eventual loss of large sections of reed cover.

In response to the alarming prevalence and severity of reed die-back, the European project EUREED was initiated (van der Putten, 1997; Brix, 1999). The project’s objective was to analyse the mechanisms regulating the growth dynamics and stability of reed-dominated ecosystems, develop models and predictions of the impact of human activities and climate change, and devise management strategies for reed die-back (van der Putten, 1997; Brix, 1999). The project identifies a number of factors contributing to the dieback of *Phragmites* in Europe, including eutrophication, water management practices, genetic diversity constraints, mechanical disturbances, pollution and climate change. It seems important that these factors are addressed through integrated management strategies if reed ecosystems across the continent are to be conserved and restored (van der Putten, 1997; Brix, 1999, 1999; Čížková et al., 2000).

Hungarian lakes have not been exempt from the die-back tendency, as Lake Fertő and other lakes and wetlands in Hungary experienced significant reed disappearance during the same period (Dinka et al., 2010; Tóth, 2016). Human activities, including water regulation, urbanisation and inadequate reed management practices, have been identified as contributing factors for Hungarian lakes too. The loss of reed stands in these areas has not only resulted in a reduction in habitat availability for a diverse range of species, thereby impacting the ecosystem services they provide, but it remains a significant challenge for the conservation of European wetlands. This research aimed to understand changes in photosynthetic traits along an ecological gradient and developmental stages within a season. This study proposes a hypothesis that differences between the studied reed stands (ecological gradient) will have a significant effect on the values (a^1^) and plasticity (a^2^) of the studied photosynthetic traits. It further hypothesised that seasonal changes (phenological sequence) will have a significant effect on the values (**b^1^**) and plasticity (**b^2^**) of the studied photosynthetic traits.

## 2 Materials and methods

### 2.1 Study area

**Figure 1:**
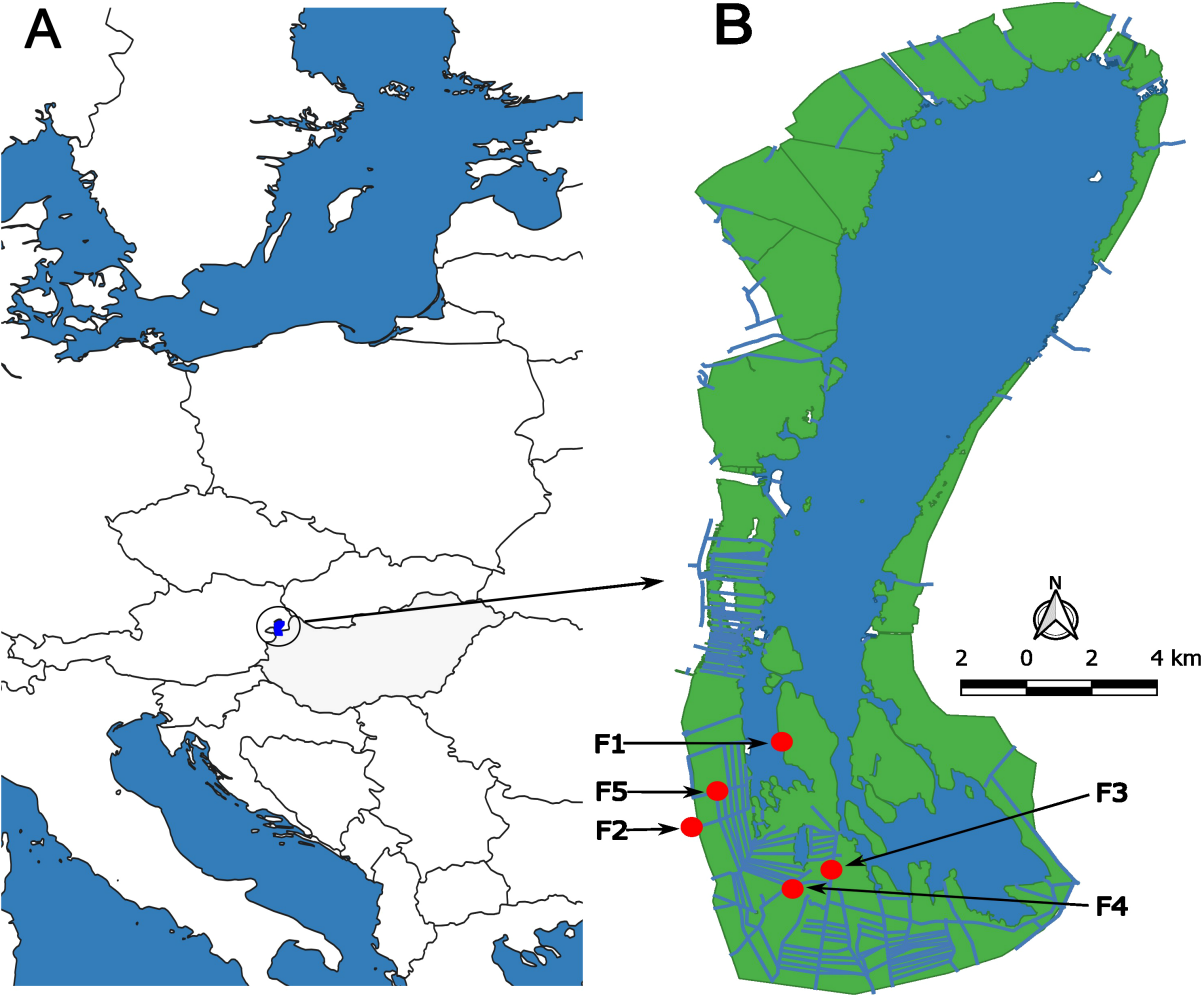
**(A)** Location of Lake Fertő (blue polygon) in Central Europe between Hungary (grey colour) and Austria. **(B)** Position of sampling points in the reed beds (green colour) of Lake Fertő. The numbering indicates the degree of degradation of the reed stands: F1 - stable site, F2 - terrestrial stable site, F3 - moderately degraded site, F4 - severely degraded site, F5 - dieback site. The artificial canal structure of the wetland is indicated by blue lines within the green reed stands.

Lake Fertő/Neusiedl is a large water body (309 km^2^) on the border of Hungary and Austria (N47.71, E16.73 - **Figure 1**). It is an endorheic lake with a relatively small catchment area of 1120 km². The lake is shallow: the average depth is 0.7 metres, while the average depth of the pelagic parts is 1.4 metres. Lake Fertő can be divided into two distinct parts: the pelagic (open water) zone and the wetland area; the water quality and environmental conditions in each zone are unique. The 85% of the Hungarian part of the lake is covered with reeds, although the overall coverage of the lake is lower (55% or about 170 km^2^). Over time, the ecological status of the reed beds has deteriorated, particularly in the Hungarian section (Dinka et al., 2004, 2010). This deterioration has been caused by both natural and anthropogenic factors. Natural causes include senescence (most reed stands are more than a decade old and may be subject to dieback), zonation related to water depth (areas of waterlogged reed stands without direct freshwater inflow often have high dissolved organic matter content) and successional changes (drying up of parts of the wetland and transition to grassland). However, anthropogenic factors such as inappropriate reed management practices and infrastructure development are likely to be the main drivers of this degradation.

Over the past 20 years, local water authorities have attempted to rehabilitate the reed stands by reconstructing canals within the wetlands to improve water supply to isolated reed stands (**Figure 1**). Unfortunately, the technology used in these reconstructions has been ineffective. The combination of these factors has resulted in significant habitat variability, with reed stands in the Hungarian part of Lake Fertő now showing varying degrees of degradation.

The aim of this manuscript is not to detail the factors causing this degradation, but rather to quantify its effects using *Phragmites* morphology as a proxy to encapsulate the effects of degradation. Although the quality of the reed beds has changed over the study period, the data presented are of scientific value not only at a local scale but also to macrophyte ecologists worldwide.

### 2.2 Macrophyte morphology and study site characterisation

To accurately categorise the selected sites ecologically, the morphological characteristics of the reed plants at each site were assessed during the peak vegetation period of the previous year (August 2019) and used as a proxy to describe the degree of degradation at these sites. At each sampling site 15 *Phragmites* plants were randomly collected cutting them either at water surface or sediment level. Stem height was determined from cut surface to the tip of the top leaf with a measuring tape. Water depth was measured at each site and added to plant height. Diameter in the middle of the basal internode of each reed stem was measured with a vernier calliper, green and dry leaves of each plant were counted, number of nodes was determined. Plant density was measured three times at each site using a 50 x 50 cm quadrat. The initial point of quadrat placement was randomly selected and subsequent quadrats were placed at 6 m intervals along a marked rope. All green *Phragmites* plants within each quadrat were counted.

Based on these data and experience of previous years reed stands of Lake Fertő were divided into 5 degradational categories (F1-F5) described as follows:

- The stable reed stand (F1) in Lake Fertő was highly monospecific, with vegetation that was tall, dense, and homogeneous (**Figure 1**). The stand was continuously exposed to wave action. The water depth on the water side of the stand ranged from 50 to 100 cm, and new reed vegetation grew vigorously along the water’s edge.
- The sampling site at the terrestrial edge of Lake Fertő’s wetland (**Figure 1** - F2) contained approximately 5-10% non-*Phragmites* species. The reed plants within this stand exhibited significant height, density, and uniformity. The water depth throughout the extent of the reed stand at the sampling site was uniform, ranging from 10 to 30 cm.
- The mildly degraded sampling site F3 was located in the centre of the wetland of Lake Fertő and was characterised by its monospecific nature, consisting mainly of *Phragmites* species (**Figure 1**). The reed plants within this stand exhibited remarkable height and density. However, small patches with no reed growth were observed in the otherwise homogeneous reed stand. The landscape was further marked by the construction of water management canals by the water authorities around the sampling site. At the sampling site, the reed stand maintained a consistent water depth of 0-20 cm throughout its extent.
- The moderately degraded sampling site F4 was situated in the centre of the wetland of Lake Fertő and had a species composition of 5-10% other than *Phragmites* (**Figure 1**). Within this stand, the reed plants were smaller and thinner, and the area was less densely populated, with frequent 10-20 square metre patches without vegetation. Water management canals were also present in the vicinity of the site. The water depth at the site ranged from 20-40 cm.
- The die-back reed stand of the wetland of Lake Fertő (**Figure 1** - F5) was result of a high level ecological degradation. Approximately 70% of the area was without any vegetation (0 plants m^-2^), while the remaining area was covered by 0.1-0.5 m^2^ patches of clumped reeds of extreme density of approximately 300-400 individuals per square metre. The reed plants within these patches were small and thin. Near the sampling site, artificial canals were reconstructed 10 years ago to rehabilitate the area, and the water depth remained consistently low, ranging from 0 to 30 cm throughout the affected area.

### 2.3 Macrophyte photophysiological measurements

Using the above site categorisations, chlorophyll fluorescence parameters were measured in 2020 and 2021 using a chlorophyll fluorometer (PAM-2500, Heinz Walz GmbH, Germany) between 9:00 and 15:00. Measurements were performed at least once a month between April and October. Plants were randomly selected using a marked rope with evenly spaced knots at 3 metre intervals, and a plant was selected nearest to the knot. However, preference was given to selecting average looking, intact plants. For sites F1-F4, measurements were taken along the waterward part of the reed stand, ~4 metres into the stand. At site F5, the reeds were clumped at varying distances, so it was decided to select the six closest clumped reeds within a manageable distance (less than 30 by 30 metres). The study areas at each site were deliberately minimised to reduce environmental variability and ensure more uniform conditions. At each site chlorophyll fluorescence measurements were made on the youngest, largest intact leaves. During this, light response curves [i.e., the electron transport rate (ETR) of the photosystem II (PSII) as a function of photosynthetically active radiation (PAR)] were measured. After dark adaptation (20 minutes), emitted initial fluorescence yield (F_o_) and maximal fluorescence yield (F_m_) resulting from a pulse of a saturated light (630 nm, intensity 3000 μmol m^−2^ s^−1^) were determined. From these, the photochemical PSII efficiency (F_v_/F_m_), coefficient of photochemical quenching (qP), and coefficient of non-photochemical quenching (qN) were calculated (**Table 1**). The measured leaves were exposed to 11 actinic lights for a duration of 15 seconds, at 630 nm, with an intensity of between 5 and 787 μmol m^−2^ s^−1^, and the ETR values were measured after each illumination step with a new pulse of saturated (3000 µmol m^-2^ s^-1^) light. Exponentially saturating curves (Eilers and Peeters, 1988) were fit to the light response data, and the maximum ETR (ETR_max_), theoretical saturation light intensity (I_k_), and maximum quantum yield for whole chain electron transport (α) were retrieved using formula from this study (Genty et al., 1989).

**Table 1:** Fluorescence parameters derived from PAM fluorometry, including equations for minimum (F_0_) and maximum (F_m_) fluorescence yields, apparent (F_s_) and maximum (F_m′_) fluorescence values, irradiance (I) and empirical absorption factor (AF=0.84).

Parameter	Name	Equation	Reference
*F_v_/F_m_*	maximum quantum efficiency of PSII	*(F_m_ − F_0_)/F_m_*	(Schreiber, 1998)
*qP*	photochemical quenching	*(F_m’_−F_s_)/(F_m’_−F_0′_)*	(Titus and Adams, 1979)
*qN*	non-photochemical quenching	*1−(F_m’_−F_0’_)/(F_m_−F_0_)*	(Titus and Adams, 1979)
*ETR*	electron transport rate	*(F_m’_−F_s_)/(F_m’_)·I·AF·0.5*	(Schreiber et al., 1995)

Further details can be found in the cited literature.

### 2.4 Statistics

Several statistics were used in this study using R statistical software (R Development Core Team, 2012). Descriptive statistics, including means, standard deviations and ranges, were calculated to provide a general overview of the data. One-way ANOVA was used to compare the means of photosynthetic traits between reed stands classified into different degradation levels (F1-F5). The assumptions of normality and homogeneity of variances were tested using Shapiro-Wilk and Levene’s tests. Two-way ANOVA was used to analyse the effects of two factors (e.g. degradation level and seasonality) on photosynthetic traits. The assumptions of normality and homogeneity of variances were also tested using Shapiro-Wilk and Levene’s tests. Pearson product-moment correlation was used to examine relationships between photosynthetic traits and their plasticity, and between site degradation eigenvalue and month of the year, assuming normality of residuals (tested with the Shapiro-Wilk test) and homoscedasticity (checked visually with residual plots). Where assumptions of ANOVA or Pearson correlation were violated, log transformations were used to normalise the data and stabilise variances.

The plasticity of photophysiological responses in this study was quantified using the coefficient of variance (CV), which is a statistical measure of the relative variability of a parameter. The CV expresses the extent of variability in relation to the mean of the parameter. For each photophysiological trait measured at a specific spot (F1-F5) on a given date (month), the CV was calculated as follows:


$$CV=\left(\frac{\sigma }{\mu }\right),$$

where σ - is the standard deviation of the measured parameters, while µ - is the mean value of the measured parameter (Schlichting and Levin, 1984).

## 3 Results

### 3.1 Site characterisation

**Figure 2:**
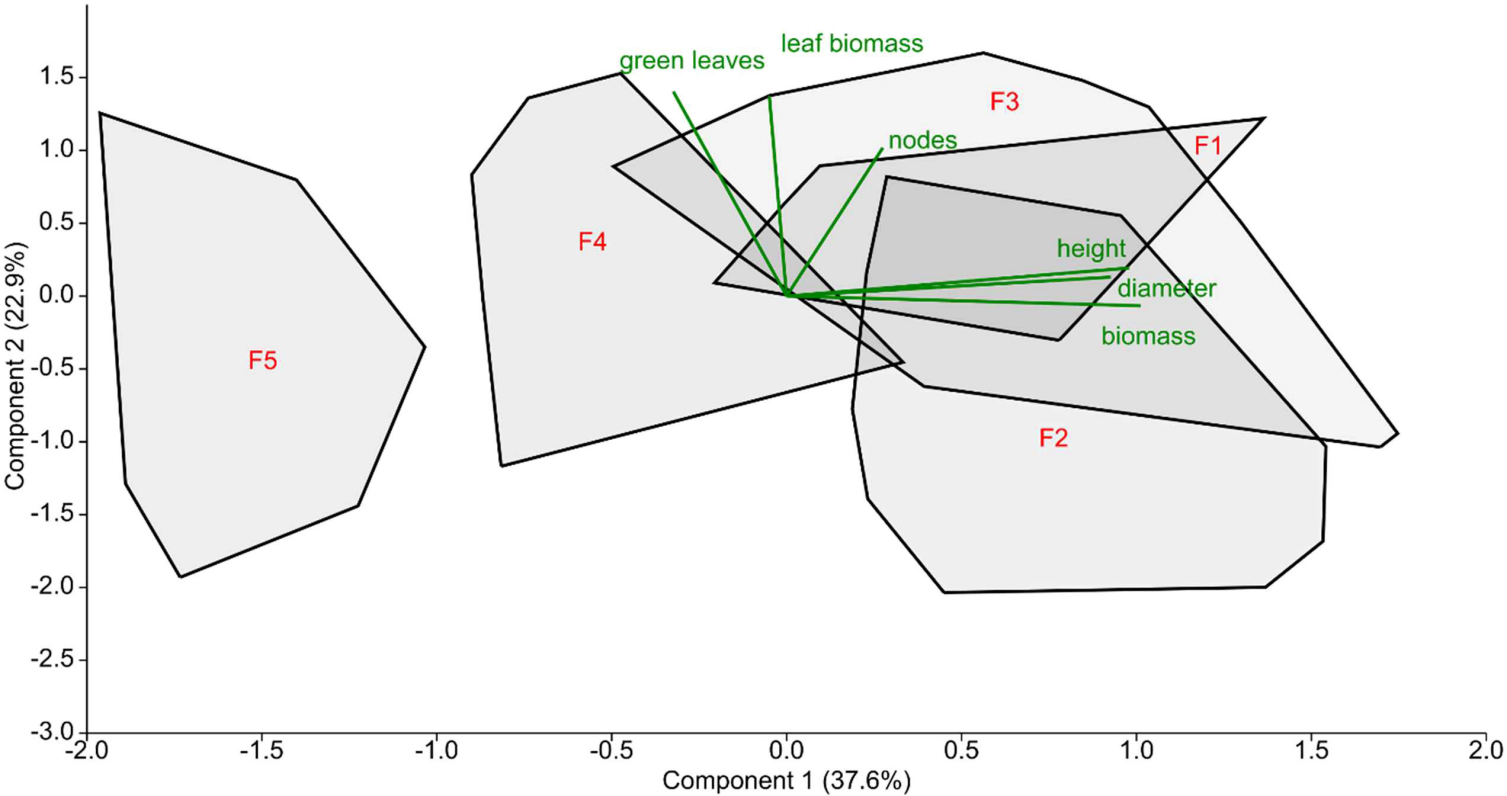
Principal components analysis of morphological traits of studied reed stands from August of 2019. Convex hulls show the data distribution at each study site (F1 – stand is a stable stand, F2 – semi-terrestrial stand, F3 and F4 – degrading sites, F5 – is the die-back site), percentage of explained variation are shown on the graph axis, biplots (green lines) represent a projection of the original, morphological axes (variables) onto the scattergram.

The selected reed stands of Lake Fertő were arranged along a degradation gradient based on their morphological parameters (**Table 2**; **Figure 2**). Some of the recorded traits showed clear signs of *Phragmites* degradation, especially height, diameter, biomass and leaf biomass (**Table 2**), while other parameters were not affected by degradation. The changes in morphological parameters across the degradation gradient were not equidistant or linear, as F1, F2 and F3 showed significant similarity, whereas F4 and F5 were more affected by degradation. For example, the average stem height of reeds in F1, F2 and F3 stands was 326, 295 and 280 cm respectively, while plants in degraded (F4) and dieback (F5) stands were 34% and 48% smaller than those in F1 (**Table 2**). Similar trends were observed for basal diameter, number of nodes, and average plant biomass (**Table 2**).

**Table 2:** Biometric properties of *Phragmites australis* plants from the studied reed stands in Lake Fertő in August of 2019 (n=15).

	Water depth (cm)	Height (cm)	Diameter (mm)	Green leaf	Node number	Biomass (g)	Leaf biomass (%)
F1	74 ± 13	252.7 ± 17.1	7.2 ± 1.8	11.6 ± 0.6	20.6 ± 5.4	23.5 ± 3.4	32.1 ± 0.8
F2	27 ± 34	268.6 ± 44.5	7.7 ± 0.6	9.5 ± 2.7	19.7 ± 8.1	24.1 ± 5.2	27.5 ± 5.0
F3	11 ± 7	269.9 ± 57.5	7.6 ± 1.1	12.4 ± 4.3	22.0 ± 4.9	29.4 ± 11.6	31.4 ± 3.1
F4	23 ± 18	190.2 ± 16.7	7.1 ± 0.4	12.5 ± 3.8	19.1 ± 5.2	14.3 ± 3.9	32.1 ± 5.8
F5	6 ± 8	163.05 ± 8.1	5.1 ± 0.6	11.0 ± 2.6	16.9 ± 4.5	9.6 ± 2.0	29.6 ± 4.9
	ANOVA						
	F	30.6	15.8	2.4	1.6	25.1	3.2
	P	<0.001	<0.001	0.059	0.180	<0.001	0.019

F1 and F2 are stable aquatic and terrestrial stands, F3 and F4 are stands at different degree of degradation, while F5 is a die-back stand.

The variation in plant density between sampling sites also reflected the response of plants to the different ecological conditions of their stands. Higher densities (F1 = 67 plants m^-2^, F2 = 79 plants m^-2^) indicated optimal conditions and more established stands, whereas the lower densities of site F3 (61 plants m^-2^) indicated an established, but slightly disturbed and less dense reed stand compared to F1 and F2. The F4 and F5 sites (48 and 7 plants m^-2^, respectively) indicated that reed plants were responding to developing challenges and significant ecological degradation.

The dry biomass of green *Phragmites* plants in the wetlands of Lake Fertő varied significantly between 0.2 and 1.9 kg m^-2^. Higher biomass values (F1 = 1.6, F2 = 1.9, F3 = 1.8 kg m^-2^) indicate more productive areas in the favourable conditions of the stable stands, while in the degraded and dieback areas of F4 and F5 the lower values (0.7 and 0.2 kg m^-2^, respectively) indicated a substantial decrease in the overall productivity of the plants due to the suboptimal conditions.

The principal component analysis showed that the reed stands at sampling sites F1, F2 and F3 were grouped together, while F4 and F5 were separated from this group to varying degrees, although only sampling site F5 exhibited difference (**Figure 2**). The eigenvalues using Component 1 of the PCA of each site with the assigned degradation level (F1 - stable reed stand, etc., F5 - dying reed stand) were used in the correlation analysis (**Figure 2**; **Table 3**).

**Table 3:** Pearson product moment correlation (correlation coefficient and its significance - *r^p^*) between the studied photophysiological parameters (data) of *Phragmites australis* plants and their variability (cv) with the site eigenvalue (site, n=84) and the month of year (n=12).

	Data		CV	
	Site	Month	Site	Month
α	0.366	-0.74	-0.767	0.758
ETR_max_	0.892	-0.968	0.713	0.691
Ik	0.486	-0.978	0.872	0.964
qP	0.484	-0.927	0.718	0.898
qN	-0.783	0.891	0.539	-0.841
F_v_/F_m_	-0.090	-0.809	0.375	0.866

The shown parameters are the maximum electron transport capacity (ETR_max_), the theoretical saturation light intensity (I_k_), the non-photochemical quenching (qN), the photochemical quenching (qP), maximum quantum efficiency of PSII (F_v_/F_m_) of *Phragmites australis* plants. The significance of the correlations.

### 3.2 Photophysiology data

**Figure 3:**
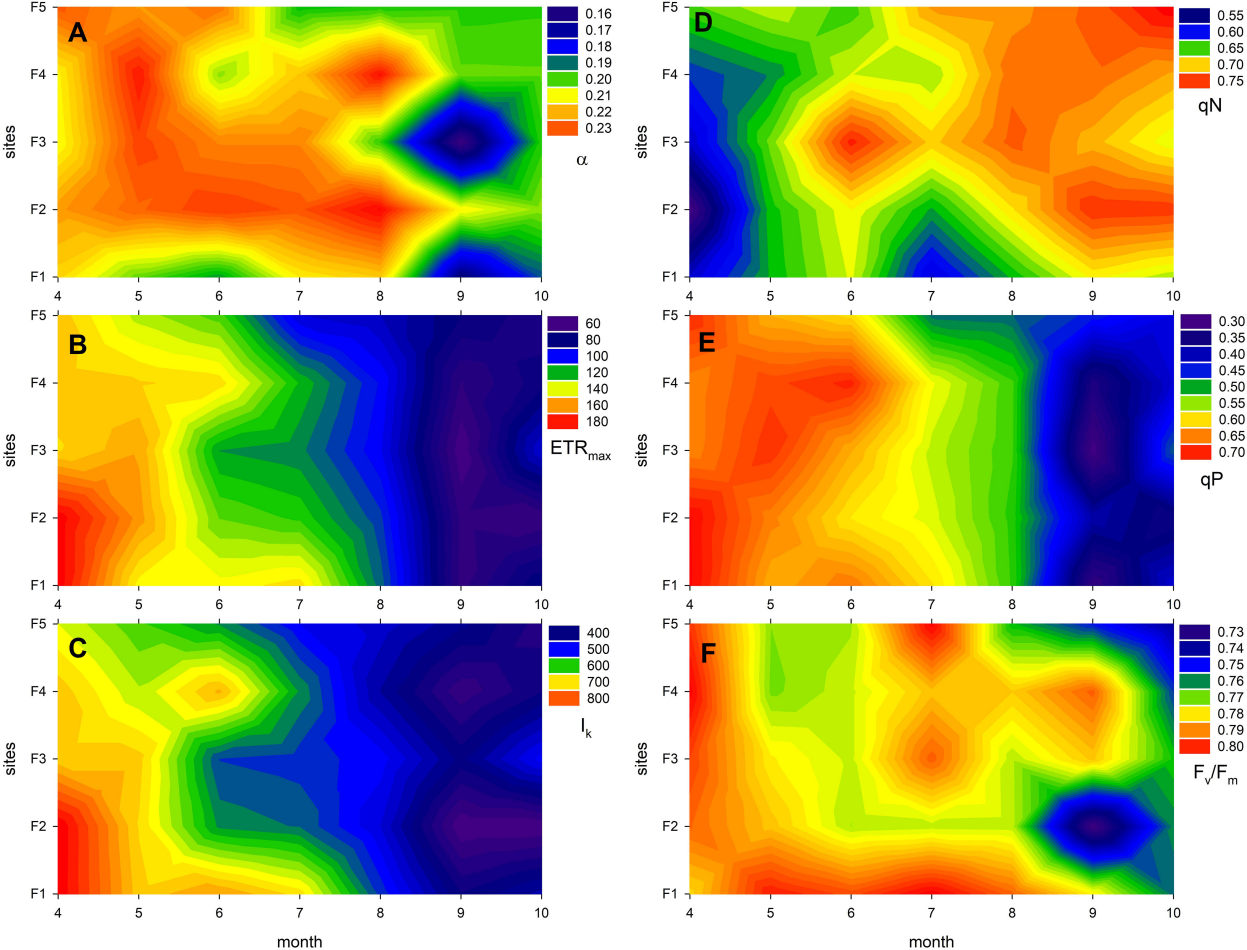
Expression of photophysiological traits of *Phragmites australis* in Lake Fertő during the study period (2020-2021) at sites with different degrees of degradation (F1 - stable to F5 - dying reeds). The figure shows the monthly averages of twelve measurements per site. Panel **(A)** shows the photosynthetic efficiency of photosystem II (α, the initial slope of the light curve), **(B)** shows the maximum electron transport rate (ETR_max_), **(C)** shows the light saturation point (I_k_, the light intensity at which ETR_max_ is reached), **(D)** shows the non-photochemical quenching (qN, indicating thermal dissipation of excess light energy), **(E)** shows photochemical quenching (qP, reflecting the fraction of open reaction centres), and panel **(F)** shows the maximum quantum yield of photosystem II (F_v_/F_m_, a measure of photosynthetic performance and stress level).

The photophysiological traits of *Phragmites* plants were significantly affected by both their ecological (level of degradation) and phenological status, as well as their interaction (**Figure 3**; **Tables 3**, **4**). The studied photophysiological traits described the potential photosynthetic efficiency of reed plants from different stands as it was affected by the process of reed degradation: minor, though significant effects were connected to the degradation of the reed stands (**Figure 3**; **Supplementary Figure S1**; **Table 4**), suggesting a reduction in the efficiency of light absorption and electron transport and resulting in lower potential photosynthetic rates. Parallel to this, gradual increase in qN (non-photochemical quenching) showed the increased disbalance in photochemical and non-photochemical processes of the degraded stands, for example increased intensity of photoprotection processes (**Figure 3D**).

**Table 4:** Two-way ANOVA of photophysiological traits of *Phragmites australis* plants in the wetlands of Lake Fertő.

	Site	Month	Interaction
α	8.17^***^	11.88^***^	4.12^***^
ETR_max_	8.30^***^	88.98^***^	2.79^***^
I_k_	10.13^***^	59.56^***^	2.15^**^
qP	3.20^*^	90.60^***^	2.16^**^
qN	7.52^***^	14.58^***^	1.92^*^
F_v_/F_m_	4.15^**^	9.35^***^	1.91^*^

F-test values and significance are shown (F*^p^*). The measured traits are the maximum electron transport capacity (ETR_max_), the theoretical saturation light intensity (I_k_), the non-photochemical quenching (qN), the photochemical quenching (qP), maximum quantum efficiency of PSII (F_v_/F_m_) of *Phragmites* plants. The significance of the correlations: ^*^ *p*<0.05, ^**^ *p*<0.01, ^***^ *p*<0.001.

Main factors: sites (degradation level) and months.

The photophysiological parameters also exhibited significant seasonal changes that exceeded the effects caused by degradation (**Figure 3**; **Supplementary Figure 2**; **Tables 3**, **4**). Except for qN, all major photophysiological parameters started from high values in spring and gradually decreased towards the end of the season, as seasonal senescence caused a decrease in light absorption and electron transport efficiency of the plants (**Figures 3B, C, E**). Specifically, ETR_max_, I_k_ and qP decreased by 57%, 51% and 45%, respectively, regardless of the ecological status of the reed stands (**Figures 3B, C, E**; **Supplementary Figure 2**; **Tables 3**, **4**). The likelihood of experiencing oxidative stress increased with plant age, resulting in a decrease in the maximum quantum efficiency of PS II (F_v_/F_m_) from 0.80 to 0.76 (a 5% decrease) (**Figure 3F**). To protect against this oxidative stress and to mitigate the excess energy absorption resulting from the decreased light absorption efficiency, non-photochemical quenching (qN) increased by approximately 23% (**Figure 3D**).

### 3.3 Plasticity of photophysiological data

**Figure 4:**
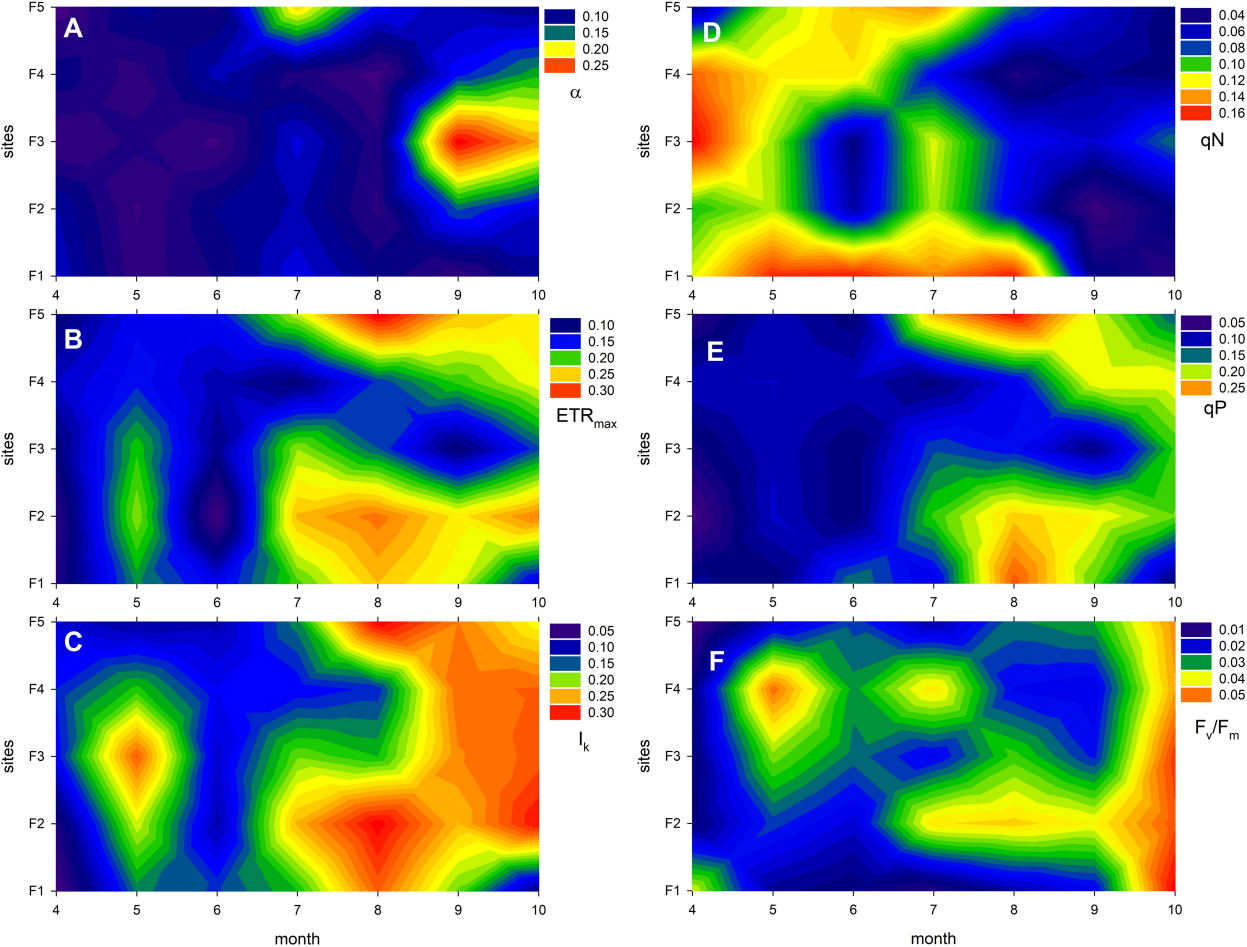
Plasticity of photophysiological traits of *Phragmites australis* in Lake Fertő during the study period (2020-2021) at sites with different degrees of degradation (F1 - stable to F5 - dying reeds). Plasticity was calculated as coefficient of variation (cv). Figures show average of 2 sets of measurements (2020 and 2021) per site per month. Panel **(A)** shows the cv of the photosynthetic efficiency of photosystem II (α, the initial slope of the light curve), **(B)** shows the cv of the maximum electron transport rate (ETR_max_), **(C)** shows the cv of the light saturation point (I_k_, the light intensity at which ETR_max_ is reached), **(D)** shows the cv of the non-photochemical quenching (qN, indicating thermal dissipation of excess light energy), **(E)** shows the cv of photochemical quenching (qP, reflecting the fraction of open reaction centres), and panel (F) shows the cv of the maximum quantum yield of photosystem II (F_v_/F_m_, a measure of photosynthetic performance and stress level).

Plasticity of the studied photophysiological traits (**Figure 4**; **Supplementary Figure S3**; **Table 5**) were not affected by the different degradation level of the reeds stands. Generally, sites F1, F2 and F3 exhibited the highest variability in most cases, while plants in the degraded reed stands (F4 and F5) had the lowest variability (**Figure 4**). It is worth noting that ETR_max_ and I_k_ exhibited the highest variability overall, at approximately 0.3, while F_v_/F_m_ showed the lowest variability, at approximately 0.05.

**Table 5:** Two-way ANOVA of plasticity of photophysiological traits of *Phragmites australis* plants in the wetlands of Lake Fertő.

	Site	Month
α	0.51	1.26
ETR_max_	0.55	3.57*
I_k_	1.33	3.12*
qP	0.32	2.87*
qN	1.74	4.85*
F_v_/F_m_	1.73	5.01*

Plasticity was calculated as coefficient of variation. Main factors: sites (degradation level, n=84) and months (n=12). F-test values and significance are shown (F^P^). The measured traits are the maximum electron transport capacity (ETR_max_), the theoretical saturation light intensity (I_k_), the non-photochemical quenching (qN), the photochemical quenching (qP), maximum quantum efficiency of PSII (F_v_/F_m_) of *Phragmites* plants. The significance of the correlations: ^*^P<0.05.

The plasticity of the photophysiological traits mostly showed an increasing seasonal trend (**Figure 4**; **Supplementary Figure S4**; **Table 5**). In most cases, trait variability within the season nearly doubled, although the increase in photochemical quenching and the maximum quantum efficiency of PS II was even higher (qP – 172%, F_v_/F_m_ – 143%). The plasticity of non-photochemical quenching (qN) decreased, indicating a significant reduction in variability of this parameter by the end of vegetation season (**Figure 4D**). In the background of majority of temporal variability were the seasonal changes of ETR_max_, qP, I_k_ and qN (**Supplementary Figures S5**–**S9**). The stability in annual mean photophysiological traits is shown on **Supplementary Figure S10**.

## 4 Discussion

The symptoms of reed dieback around the world are very similar, i.e. retreat from deep water, increased inhomogeneity and clumping, reduction in stem density, size and diameter, and premature senescence of plants (Armstrong et al., 1996; van der Putten, 1997; Armstrong and Armstrong, 1999; Brix, 1999; Tóth, 2016). In this work, the studied reed stands of Lake Fertő were categorised into stages of degradation based on their morphological appearance, plant density and stand homogeneity. The result of the categorisation was not equidistant, as e.g. the stable (F1 - deep water stand and F2 - terrestrial stand) and the moderately degraded (F3) reed stands were somewhat similar and were grouped in a related ecotype. The F3 stand provided an excellent example of the resilience of *Phragmites*, i.e. how reed stands can recover under the right conditions: the previously degraded (inhomogeneous, smaller) F3 reed stand changed its appearance due to the low water levels of Lake Fertő in 2020 and 2021. This led to an improvement of the ecological conditions in the surroundings of F3 and consequently to an improvement of the photophysiological parameters measured. Although these environmental conditions led to an improvement in the moderately degraded reed stands, the degraded (F4) and dying (F5) stands were not affected and showed no signs of regeneration in 2020 and 2021.

Along the established ecological gradient, photophysiological traits of *Phragmites* changed in a well-defined pattern making a^1^ hypothesis supported. The differences between sites were significant, but not equidistant, thus no prominent trends along the studied ecological gradient were identified, except for maximum electron transport capacity (ETR_max_). This apparent stability of photophysiological traits and their independence from reed stand degradation may be due to the large genetic variation of the *Phragmites* (Lambertini et al., 2008; Engloner and Major, 2011; Eller et al., 2017) paired with a high trait plasticity of plants (Clevering et al., 2001; Eller and Brix, 2012; Tóth, 2016), while data of the research suggest also a compensatory effect of phenological changes in reed populations.

On the contrary, notable seasonal variations in the photosynthetic traits were recorded (b^1^ - supported), and these trends were consistent both in degraded and stable reed stands. Along the growing season, significant decrease (or increase in the case of non-photochemical quenching - qN) in monthly averages, regardless of degradation level were observed, suggesting a universal response of reed photophysiological traits to changing seasonal environmental conditions. Although *Phragmites* plants in the Fertő wetlands have been exposed to very similar dynamic changes in environmental conditions from April to October for millennia, the autumn changes cause stress that is compensated by adjustments in photosynthetic parameters in all reed stands regardless of the degree of degradation. The pattern of changes may be driven by seasonal shift of environmental factors such as temperature, light quantity and quality, etc., which have a strong influence on photophysiological traits.

Despite differences in habitat quality and structural characteristics between degraded and stable stands, reed plants of Lake Fertő exhibited considerable and comparable photophysiological plasticity (a^2^ – not supported), allowing them to adapt to different environments and maintain a relatively constant level of photosynthetic performance under changing ecological conditions. The photophysiological plasticity of *Phragmites* plants often involves mechanisms such as altering photosynthetic rates and/or modulating enzyme activities to optimise resource use efficiency (Lessmann et al., 2001; Pagter et al., 2005; Tóth, 2016, 2018). In addition, it cannot be excluded that genetic diversity within the reed population of Lake Fertő may increase resilience by providing a wider range of adaptive traits. This genetic variation allows some clones to possess traits that confer resilience to specific environmental stressors associated with degradation, such as changes in sediment redox potential, permanent inundation, or other stresses.

A significant increase in the plasticity of photophysiological parameters with seasonal senescence in *Phragmites* plants in Lake Fertő suggests a dynamic response of the photosynthetic apparatus and makes the b^2^ hypothesis of this study true. Senescence is a natural physiological process in plants in which older tissues degrade or transform during the later stages of the plant life cycle (Gan and Amasino, 1997; Liu et al., 2016; Woo et al., 2019). During senescence, stochastic changes may occur in the chloroplasts and photosynthetic system of older leaves, leading to the increased plasticity in photochemical parameters observed in this study, although the nature of the observed trend in plasticity (**Supplementary Figure S4**) may imply an unknown deterministic background. This, combined with seasonal changes in environmental conditions (such as lower temperatures in autumn and changes in optical properties), may contribute to the increased plasticity observed with senescence. Meanwhile, other environmental, plant physiological, genetic and phenotypic factors that may be associated with degraded *Phragmites* stands do not significantly affect the plastic response of reed plants. This suggests a very specific mechanism of regulation of *Phragmites* plasticity, primarily driven by plant senescence and independent of the degradation status of reed stands.

## 5 Conclusion

It is consistent with the literature suggesting that certain groups of *Phragmites* are able to acclimate to specific environmental changes due to the increase of their phenotypic plasticity (Eller et al., 2017; Ren et al., 2020). The data from the study showed that the values of the studied photosynthetic traits of reed were indeed lower in the degraded and die-back sites. The observed reduction of photosynthetic trait values in degraded reed stands underlines the detrimental effects of ecological degradation on plant physiological processes. Contrary to the initial hypothesis, the analysis showed that the plasticity of the traits studied were not statistically significantly different between the degraded and more stable reed stands. The lack of significant differences in plasticity between degraded and stable reed stands suggests that phenotypic flexibility may not vary significantly with ecological stability. These findings highlight the refined responses of common reed to ecological gradients, and emphasise the importance of considering both trait values and plasticity in understanding plant responses to environmental change.

## Data Availability

The raw data supporting the conclusions of this article will be made available by the author, upon reasonable request.
