# Supplementary material for: Photosynthetic traits of Phragmites australis along an ecological gradient and developmental stages
Source: Front Plant Sci. 2025 Jan 8;15:1476142. doi: 10.3389/fpls.2024.1476142 (PMC11751706; doi:10.3389/fpls.2024.1476142)
Supplement: Supplementary file 1 [file Image1.pdf]

Supplementary Material

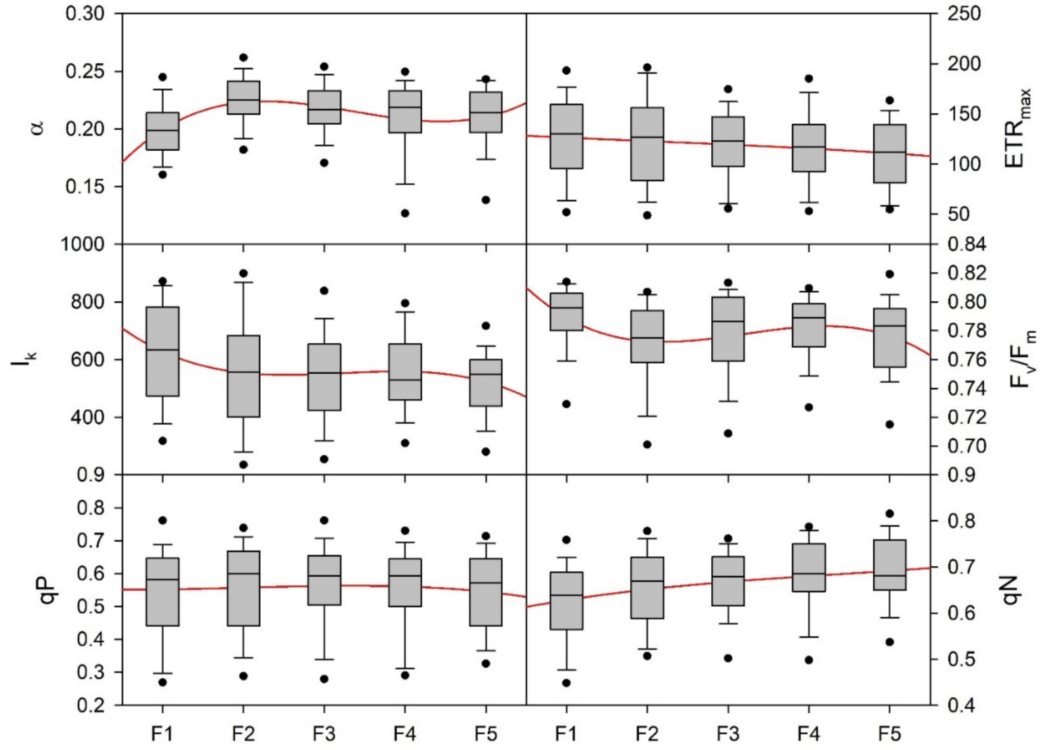

Supplementary Figure 1. Expression of photophysiological traits of *Phragmites australis* in Lake Fertő during the study period (2020-2021) in sites with different degrees of degradation (F1 – stable to F5 die-back reeds).

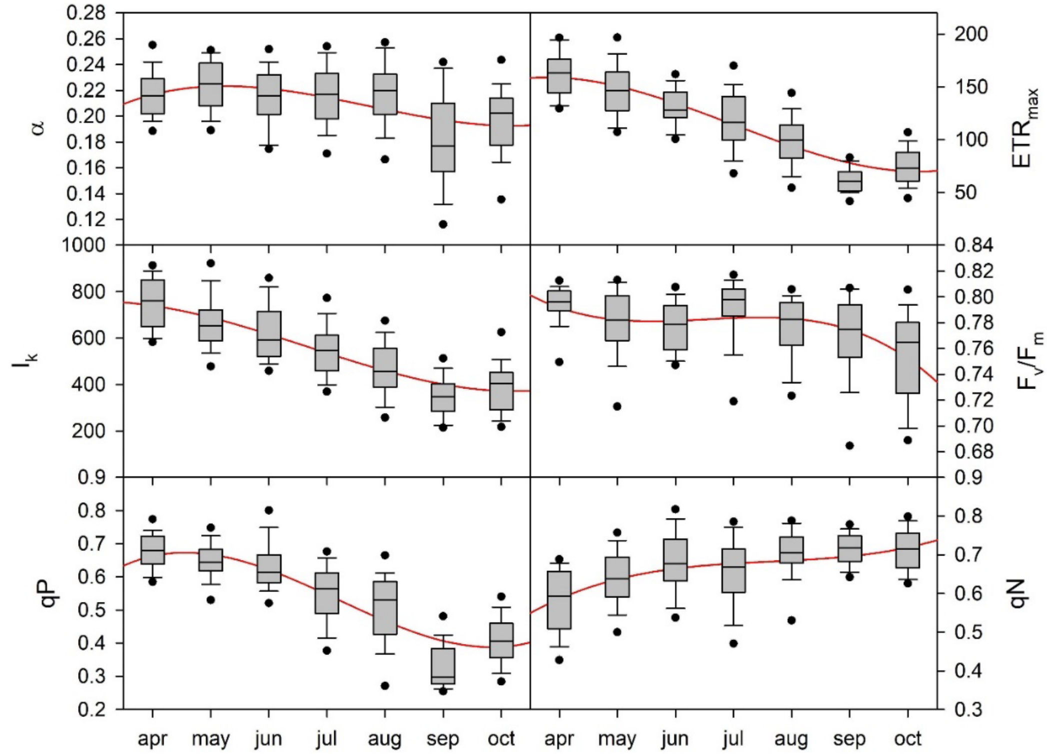

Supplementary Figure 2. Expression of photophysiological traits of *Phragmites australis* in Lake Fertő during the study period (2020-2021) along the vegetation period.

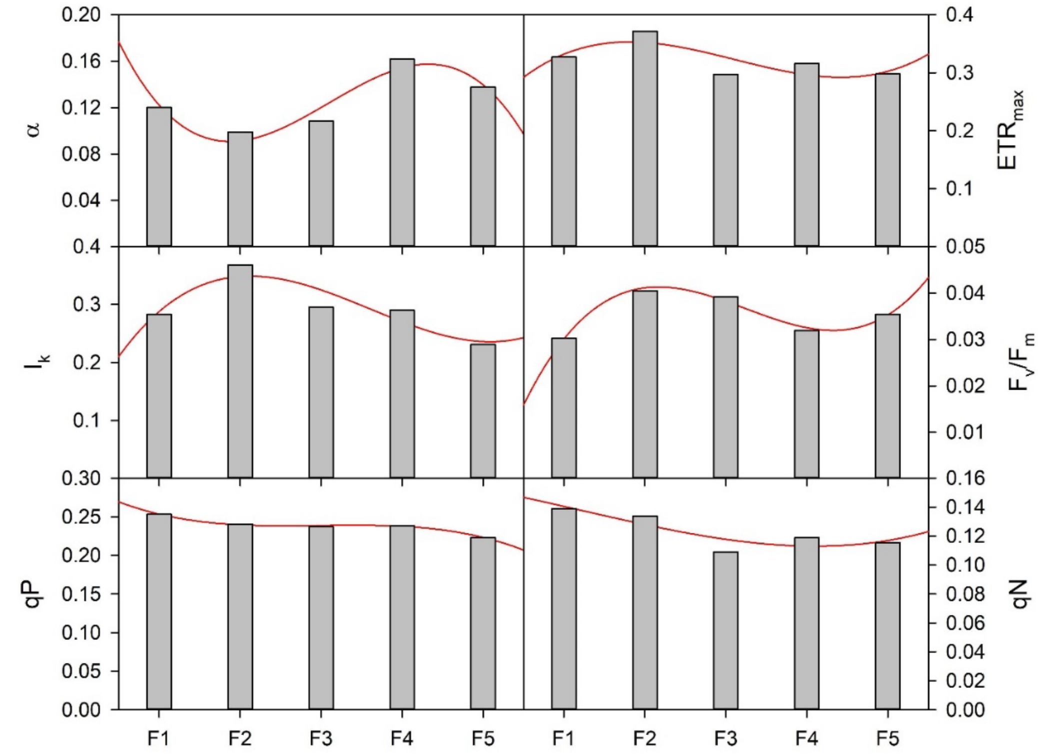

Supplementary Figure 3. Plasticity of photophysiological traits of *Phragmites australis* in Lake Fertő during the study period (2020-2021) in sites with different degrees of degradation (F1 – stable

to F5 die-back reeds).

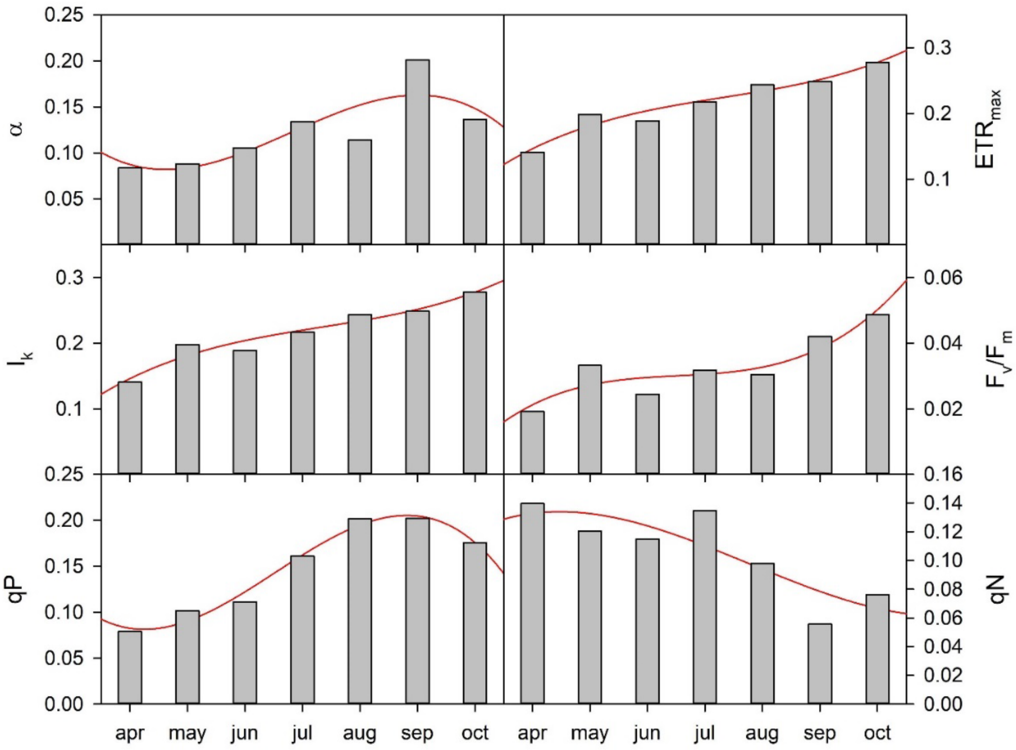

Supplementary Figure 4. Plasticity of photophysiological traits of *Phragmites australis* in Lake Fertő during the study period (2020-2021) along the vegetation period.

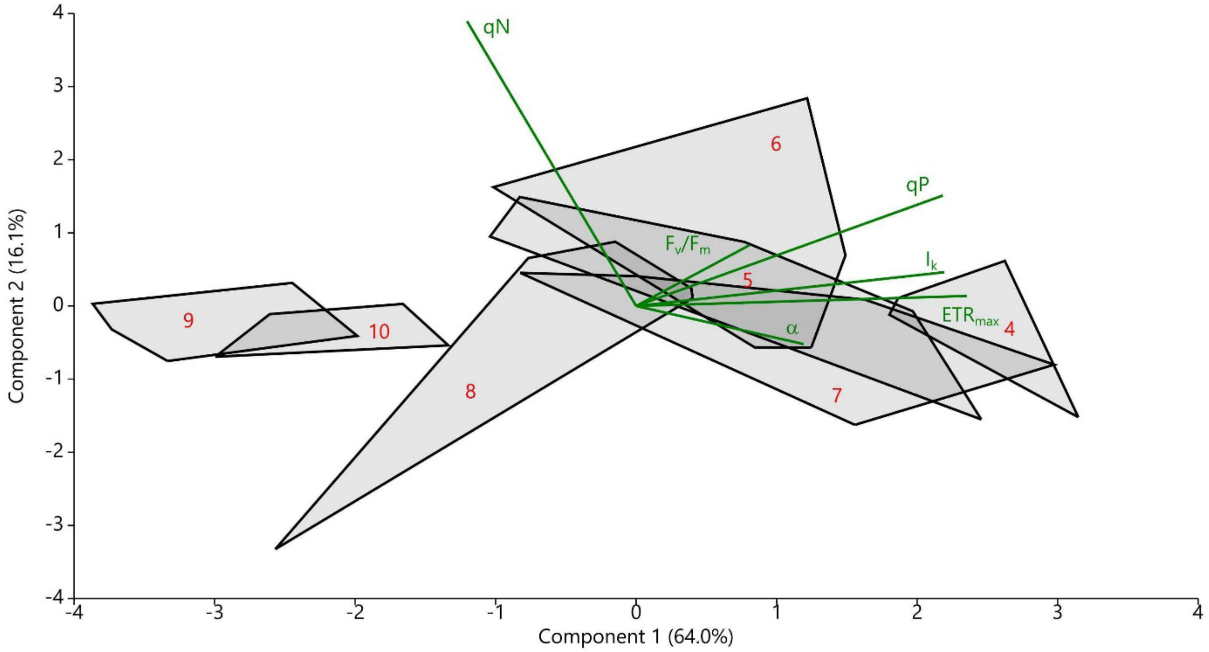

Supplementary Figure 5. Principal components analysis of photophysiological traits of the F1 reed stand in 2020 and 2021. Convex hulls show the data distribution at each month (4 - April → 10 - October), percentage of explained variation are shown on the graph axis, biplots (green lines) represent a projection of the original axes (photophysiological variables) onto the scattergram.

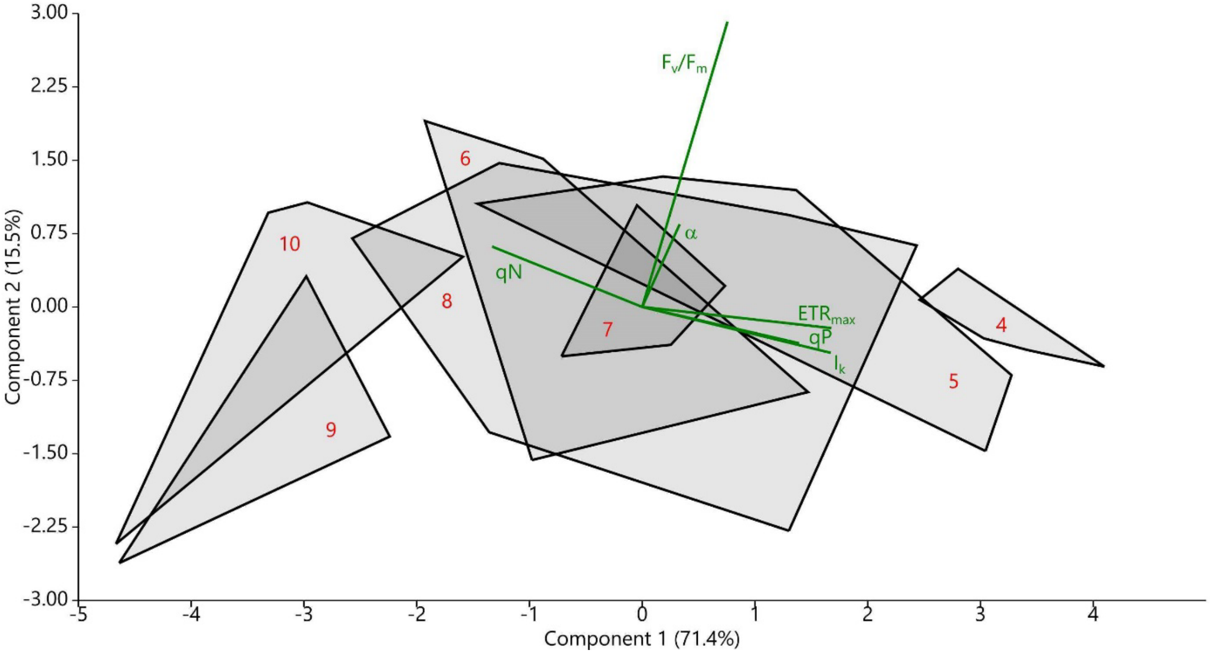

Supplementary Figure 6. Principal components analysis of photophysiological data of the F2 reed stand in 2020 and 2021. Convex hulls show the data distribution at each month (4 - April → 10 - October), percentage of explained variation are shown on the graph axis, biplots (green lines) represent a projection of the original axes (photophysiological variables) onto the scattergram.

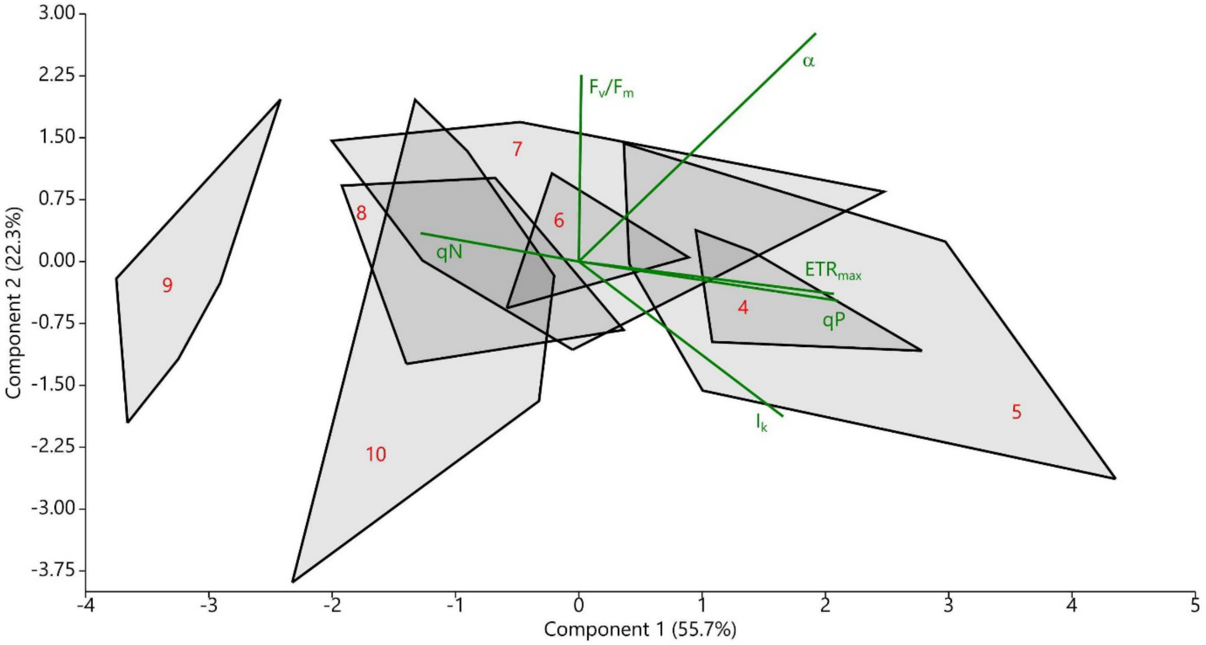

Supplementary Figure 7. Principal components analysis of photophysiological data of the F3 reed stand in 2020 and 2021. Convex hulls show the data distribution at each month (4 - April → 10 - October), percentage of explained variation are shown on the graph axis, biplots (green lines) represent a projection of the original axes (photophysiological variables) onto the scattergram.

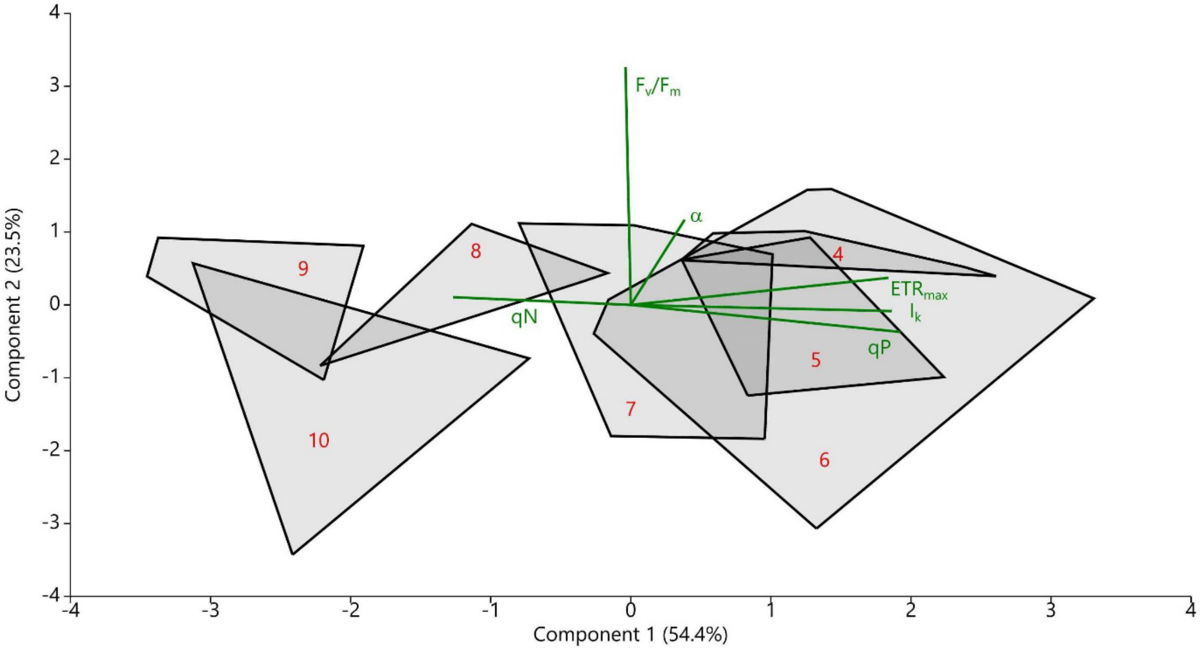

Supplementary Figure 8. Principal components analysis of photophysiological data of the F4 reed stand in 2020 and 2021. Convex hulls show the data distribution at each month (4 - April → 10 - October), percentage of explained variation are shown on the graph axis, biplots (green lines) represent a projection of the original axes (photophysiological variables) onto the scattergram.

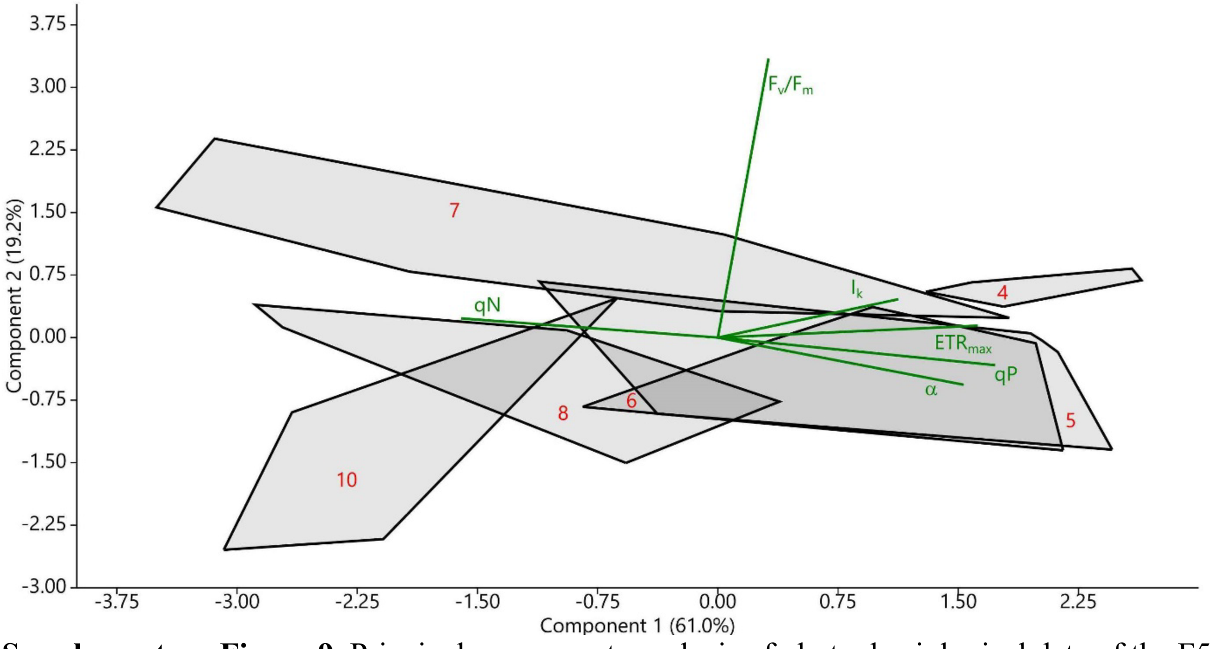

Supplementary Figure 9. Principal components analysis of photophysiological data of the F5 reed stand in 2020 and 2021. Convex hulls show the data distribution at each month (4 - April → 10 - October), percentage of explained variation are shown on the graph axis, biplots (green lines) represent a projection of the original axes (photophysiological variables) onto the scattergram.

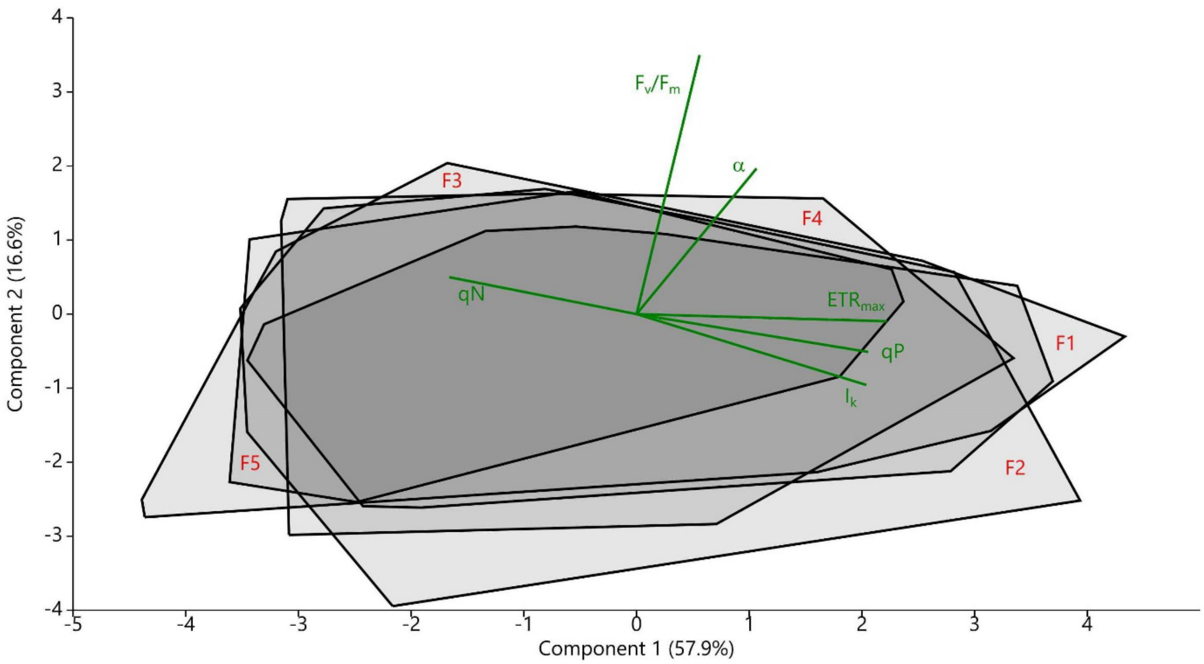

Supplementary Figure 10. Principal components analysis of photometric traits of studied reed stands in 2020 and 2021. Convex hulls show the data distribution at each study site (F1 – stand is a stable stand, F2 – semi-terrestrial stand, F3 and F4 – degrading sites, F5 – is the die-back site), percentage of explained variation are shown on the graph axis, biplots (green lines) represent a projection of the original axes (variables) onto the scattergram.
